# Decolorization of Textile Azo Dye via Solid-State Fermented Wheat Bran by *Lasiodiplodia* sp. YZH1

**DOI:** 10.3390/jof9111069

**Published:** 2023-11-01

**Authors:** Ali Borham, Mohammad K. Okla, Mohamed A. El-Tayeb, Ahmed Gharib, Hanan Hafiz, Lei Liu, Chen Zhao, Ruqing Xie, Nannan He, Siwen Zhang, Juanjuan Wang, Xiaoqing Qian

**Affiliations:** 1 Key Laboratory of Cultivated Land Quality Monitoring and Evaluation, Ministry of Agriculture and Rural Affairs, Yangzhou University, Yangzhou 225127, China; ali.borham@agr.kfs.edu.eg (A.B.); wangjuanjuan@yzu.edu.cn (J.W.); 2 Agriculture Products Safety and Environment, College of Agriculture, Yangzhou University, Yangzhou 225127, China; 3 Agricultural Botany Department, Faculty of Agriculture, Kafrelsheikh University, Kafr El-Sheikh 33516, Egypt; 4 Botany and Microbiology Department, College of Science, King Saud University, P.O. Box 2455, Riyadh 11451, Saudi Arabia; malokla@ksu.edu.sa (M.K.O.); mali5@ksu.edu.sa (M.A.E.-T.); 5 National Institute of Laser Enhanced Sciences (NILES), Cairo University, Giza 12613, Egypt; ahmgharib@niles.edu.eg; 6 Biotechnology Department, Faculty of Science, Damietta University, New Damietta 34517, Egypt; hananhafizz5@gmail.com; 7 College of Environmental Science and Engineering, Yangzhou University, Yangzhou 225127, China; ll18252788565@163.com (L.L.); zhaochen1117yz@163.com (C.Z.); 15252753630@163.com (R.X.); hnn991012@163.com (N.H.); zhangsiwen980202@163.com (S.Z.)

**Keywords:** dye removal, biosorption, Congo red, statistical optimization, Box–Behnken design, solid-state fermentation

## Abstract

Textile dyes are one of the major water pollutants released into water in various ways, posing serious hazards for both aquatic organisms and human beings. Bioremediation is a significantly promising technique for dye decolorization. In the present study, the fungal strain *Lasiodiplodia* sp. was isolated from the fruiting bodies of *Schizophyllum* for the first time. The isolated fungal strain was examined for laccase enzyme production under solid-state fermentation conditions with wheat bran (WB) using ABTS and 2,6-Dimethoxyphenol (DMP) as substrates, then the fermented wheat bran (FWB) was evaluated as a biosorbent for Congo red dye adsorption from aqueous solutions in comparison with unfermented wheat bran. A Box–Behnken design was used to optimize the dye removal by FWB and to analyze the interaction effects between three factors: fermentation duration, pH, and dye concentration. Fourier transform infrared spectroscopy (FTIR), X-ray diffraction (XRD), and scanning electron microscopy (SEM) were applied to study the changes in the physical and chemical characteristics of wheat bran before and after fermentation. An additional experiment was conducted to investigate the ability of the *Lasiodiplodia* sp. YZH1 to remove Congo red in the dye-containing liquid culture. The results showed that laccase was produced throughout the cultivation, reaching peak activities of ∼6.2 and 22.3 U/mL for ABTS and DMP, respectively, on the fourth day of cultivation. FWB removed 89.8% of the dye (100 mg L^−1^) from the aqueous solution after 12 h of contact, whereas WB removed only 77.5%. Based on the Box–Behnken design results, FWB achieved 93.08% dye removal percentage under the conditions of 6 days of fermentation, pH 8.5, and 150 mg L^−1^ of the dye concentration after 24 h. The fungal strain removed 95.3% of 150 mg L^−1^ of the dye concentration after 8 days of inoculation in the dye-containing liquid culture. These findings indicate that this strain is a worthy candidate for dye removal from environmental effluents.

## 1. Introduction

The rapid increase in industrialization and urbanization has led to the manufacture of numerous chemicals, including dyes that have been utilized in several industries, such as textiles, food, pharmaceuticals, cosmetics, plastics, and paper [1,2]. Water pollution with dyestuff prevents light from penetrating the water, directly threatening the life of aquatic creatures [3]. For human beings, a lot of synthetic dyes are toxic, mutagenic, and carcinogenic. They have a negative effect on the liver, kidneys, central nervous system, and reproductive system [3,4].

Azo dyes are widely utilized in the textile industry. They contribute to 60 to 70% of all textile industry dyes [5]. The azo dye Congo red, which is widely used in the textile and paper industries, has been found to be exceedingly carcinogenic and harmful to both humans and the environment [6]. Therefore, industrial effluents should be appropriately treated before being released into the environment to avoid environmental issues.

A number of physical and chemical techniques, including adsorption, coagulation–flocculation, photocatalytic ozonation, and inorganic catalysis, have been proposed for dye decolorization [7,8,9]. However, many industrial dyes pass through the majority of these conventional processes, and because of their high level of resistance to temperature, light, and oxidizing agents, they remain in the environment for a long period of time. Moreover, these conventional techniques tend to produce secondary pollutants and are rather expensive [10]. Consequently, it is still vital to develop advanced, economical, and environmentally friendly wastewater treatment technologies in order maintain natural resources.

Biosorption, biodegradation, and fungal remediation are widely used as a biological method for dye decolorization [11]. Physical adsorption and microbial degradation are important examples of techniques with a high potential for dye removal in water [12,13]. Adsorption has been a commonly utilized method for removing dyes and other recalcitrant compounds from effluents, due to its relatively low input, high effectiveness, and simplicity of use; therefore, adsorption has emerged as a promising alternative for wastewater decolorization [14,15].

Plant biomasses are either directly used as biosorbents to remove dyes from effluents or pretreated/modified to improve adsorptive efficacy via thermal, physical, chemical, microbial, or their combination procedures [16,17]. The combination of the adsorption and the microbial decolorization by solid-state fermentation (SSF) is an effective method for dye removal and enzyme production. Some reports suggest that the removal of dyes from textile wastewaters can be achieved using SSF techniques with agricultural wastes such rice bran, yeast biomass, and sugarcane bagasse [18,19,20].

Dye removal optimization has been performed in many previous reports to improve the efficiency of decolorization by one factor at a time (OFAT) [21,22,23] and response surface methodology (RSM) methods [24,25,26]. RSM is a collection of mathematical and statistical techniques used for studying the relationship between influencing factors and response values, and for obtaining the optimal conditions for a specific system [27,28,29]. The Box–Behnken design (BBD) is one of the most commonly used RSM methods. It allows the modeler to explore the relationships between the design variables and the response variables without having to conduct a large number of experiments, which is the main advantage of this analysis; it also has simple and efficient software operation, and high reliability and accuracy of optimization results [30,31]. BBD has been successfully used for the optimization of process parameters in dye decolorization [32,33], heavy metal biosorption [34,35], and a wide range of other field applications [36,37].

Agricultural wastes inoculated with different fungi that produce ligninolytic enzymes such as laccase, manganese peroxidase (MnP), and lignin peroxidase (LiP) have been widely used to treat recalcitrant xenobiotics in wastewater, including dyes [38,39,40,41,42]. White rot fungi (WRF) are widely used to treat recalcitrant xenobiotics in wastewater, as they are the major producer of ligninolytic enzymes [43]. 

*Lasiodiplodia* is a phytopathogenic fungus from the family *Botryosphaeriaceae*. This fungus and other fungi belonging to this family often remain latent inside their host tissues, in which they are able to establish endophytic infections for long periods [44]. Some previous reports referred to the capability of the fungus *Lasiodiplodia* to produce ligninolytic enzymes and its application in the bioremediation of different contaminants [45,46].

Dye removal using inoculation of fungal strains in agricultural wastes under solid-state fermentation conditions has previously been extensively reported [47,48,49]. However, there is little information available on the effect of SSF of agricultural wastes on dye removal efficiency in aqueous solutions.

In this study, the fungal strain *Lasiodiplodia* sp. YZH1 was isolated from the fruiting bodies of *Schizophyllum* for the first time, and used for wheat bran fermentation under SSF (FWB) as a cheap biosorbent for Congo red removal from aqueous solutions. A Box–Behnken design was used for the optimization of dye removal by FWB. The characterization of the wheat bran before and after fungal fermentation was performed. Additionally, dye removal assay by inoculation of *Lasiodiplodia* sp. YZH1 in aqueous solutions with different dye concentrations was also studied.

## 2. Materials and Methods

### 2.1. Isolation and Identification of the Fungal Isolate

The fungus used in this study was isolated from fruiting bodies of the white rot fungus *Schizophyllum*. The healthy fruiting bodies of *schizophyllum* were collected from a tree trunk on the Yangzijin campus, Yangzhou University, Jiangsu, China, then kept in a sealed plastic bag and brought immediately to the laboratory for the isolation. 

The samples were chopped into small segments (~3 mm) under aseptic conditions, their surfaces sterilized with 70% ethanol solution for 1 min, then rinsed with sterilized distilled water for 1 min. Sterilized segments were air-dried aseptically then cultured on PDA (potato dextrose agar) and incubated at 28 °C (GWP-160A; Huadeli Scientific Equipment Co., Ltd.; Hefei, China). The plates were checked daily for the growth of fungal mycelium. The hyphae were transferred to fresh PDA plates to obtain a pure culture and then maintained on PDA slants at 4 °C. 

The fungal isolate was identified by morphological traits based on microscopic features. In addition, the 18S rRNA gene sequencing technique was used for molecular identification. 

Genomic DNA was extracted using Ezup Column Fungal Genomic DNA Extraction Kit (Sangon Biotech, Shanghai, China). The 18S rRNA of the fungal strain was amplified by PCR using the universal fungal primers NS1, NS6 as presented by White et al. [50]. The amplification protocol was as follows: one cycle of initial denaturation at 95 °C for 4 min followed by 30 cycles of denaturation at 95 °C for 20 s, annealing at 58 °C for 20 s, extension at 72 °C for 1 min, and final extension at 72 °C for 10 min. The PCR product was gel-purified using SanPrep Column DNA Gel Recovery Kit and sequenced in Sangon Biotech^®^ (Shanghai, China) by using ABI^®^ 3730xl DNA sequencer (Applied Biosystems, Waltham, MA, USA).

The obtained sequence was compared with those deposited in GenBank database for significant alignments using the Basic Alignment Search Tool (BLAST) (https://blast.ncbi.nlm.nih.gov) (accessed on 12 September 2023). 

The phylogenetic analysis was conducted using MEGA software (version 11.0.13) and the neighbor-joining algorithm from 1000 bootstrap replicates [51].

### 2.2. Solid-State Fermentation of Wheat Bran

Wheat bran was selected in this study as a substrate for solid-state fermentation with the fungal isolate to assay ligninolytic enzymes and as a biosorbent for Congo red dye decolorization. 

A total of 5 g of wheat bran (obtained from Yangtai chemical Co., Ltd., Yangzhou, China) was sieved to collect 2 mm sized particles and then mixed with 5 mL of basal salt media ((NH4)_2_SO_4_, 1.4 g L^−1^; Na_2_HPO_4_·12H_2_O, 1.2 g L^−1^; KH_2_PO_4_, 0.9 g L^−1^; KCl, 0.5 g L^−1^; MgSo_4_·7H_2_O, 0.5 g L^−1^; yeast extract, 0.5 g L^−1^; and pH 6.5), then autoclaved at 121 °C for 20 min.

The SSF process was performed in Petri dishes (100 mm) as follows. A total of 10 g of the autoclaved mixer of wheat bran and the basal media was distributed in each sterilized Petri dish, then each Petri dish was inoculated at the center by one mycelial agar disk (with a diameter of ~5 mm) from the colony margin of 7-day-old culture of the fungal isolate grown on PDA plates. After that, all plates were incubated at 28 °C for 8 days. 

Fungal growth was recorded every 24 h by analyzing photographic records using the image-processing software Image J^®^ v. 1.53 (National Institutes of Health, Bethesda, MD, USA) [49,52,53,54]. The growth of the fungus was described as a fungal growth area (%) and calculated using the following equation:

(1) $$Fungal\;growth\left(\%\right)=\frac{A}{Petri\;dish\;total\;area}\times 100$$

where A is the growth area obtained by the Image J software at different days sampled (cm^2^), and the Petri dish total area is 56.7 cm^2^.

The biomass loss of the substrate was measured at two-day intervals for up to eight days by calculating the weight changes in the dry fermented wheat bran in each Petri dish for each sampling day [55]. All experiments were performed in triplicate for each sampling day using the same operational conditions.

### 2.3. Enzyme Extraction and Assay

The extracellular enzyme of laccase was determined at 2-day intervals (2, 4, 6, and 8 days after inoculation) by transferring the contents of the fermented wheat bran from each Petri dish (as described above) into a 250 mL conical flask. Then, 50 mL of 0.05 mM citric acid buffer (pH 4.8) was added into each flask and stirred on an orbital shaker (Honghua, HY-5B, Changzhou, China) at room temperature, 200 rpm for 2 h. The mixture was then primary filtered via Whatman filter paper and secondary filtered with a 0.45 µm syringe filter. The change in pH was also recorded. The fungal extract after the two-step filtration was considered as a crude enzyme and used immediately for enzyme assay.

Laccase activity was determined spectrophotometrically (INESA, 722N, Shanghai China) by recording the oxidation of 5mM 2,2′-azinobis-(3-ethylbenzothiazoline-6-sulfonic acid) (ABTS) in Na-citrate buffer (0.05 mM, pH 4.8) at 420 (ε420 = 36,000 M^−1^ cm^−1^) nm for 5 min. The assay mixture contained 100 µL of crude enzyme, 800 µL Na-citrate buffer (0.05 mM, pH 4.8), and 100 µL of 5 mM ABTS and was assayed at room temperature at OD 420 nm at 1 min intervals for 5 min [56]. Additionally, laccase activity was assayed using another substrate 2,6-Dimethoxyphenol (DMP) at OD 470 nm (ε470 = 27,500 M^−1^ cm^−1^) at 1 min intervals for 5 min [48]. The assay mixture contained 50 µL of the crude enzyme, 1000 µL Na-citrate buffer (0.05 mM, pH 4.8), and 200 µL of 10 mM DMP. One unit of enzyme activity was defined as the amount of enzyme required to oxidize 1 μmol of the substrate in 1 min. The activity was expressed as U/mL.

### 2.4. Dye Removal Assays

Dye removal efficiency was studied with 6-day-fermented wheat bran in Petri dishes by the fungal isolate *Lasiodiplodia* sp. YZH1 (denoted as FWB) in comparison with unfermented wheat bran (referred as WB). WB and FWB from each Petri dish were oven-dried at 70 °C for 48 h and ground to a fine powder and then stored at room temperature prior to use for decolorization studies.

Dye adsorption assays were performed in 50 mL Falcon tubes by transferring 250 mg of the WB or FWB and 25 mL of 100 mg L^−1^ of Congo red solution (prepared from 1000 mg L^−1^ stock solution in distilled water). Then, all tubes were shaken at 150 rpm and 25 °C using an orbital shaker for 12 h.

A 1 mL solution from each Falcon tube was withdrawn at 0, 2, 4, 6, 9, and 12 h, then centrifuged at 12,000 rpm for 10 min at 4 °C. The Congo red decolorization efficiency was determined in the supernatant spectrophotometrically by measuring the absorbance at the dye λMax (498 nm), which is the wavelength of the maximum visible absorbance of Congo red; it is referred to as dye removal percentage (%) and calculated according to Equation (2) [53,57,58]:

(2) $$Dye\;removal\;percentage\left(\%\right)=\frac{A_{0}-A_{t}}{A_{0}}\times 100$$

where A_0_ denotes the initial dye absorbance and A_t_ denotes the absorbance at the t sampling time.

### 2.5. Optimization of Dye Removal

FWB was employed to statistically analyze the interaction effects between three independent variables (factors)—fermentation duration of the wheat bran (X_1_), pH of the dye solution (X_2_), and dye concentration (X_3_)—on its dye removal percentage, and to predict the optimal conditions for dye removal using the Box–Behnken design. 

The three independent variables were studied at three levels; –1, 0, and 1 (the range and levels are listed in Table 1).

The experimental matrix was designed using the Box–Behnken design in the Design-Expert^®^ software version 13.0.5.0. The response variable (predicted response) was measured with the following second-order polynomial quadratic model equation:

(3) $$Y=\beta_{0}+\sum_{i}\beta_{i}X_{i}+\sum_{ij}\beta_{ij}X_{i}X_{j}+\sum_{ii}{\beta_{ii}X}_{i}^{2}$$

where Y is the predicted response (dye removal percentage), β_0_ is the intercept, β_i_ is the linear coefficient, β_ii_ is the quadratic coefficient, and β_ij_ is the interactive coefficient. X_i_, X_j_ represent independent variables.

The model was laboratory-validated for dye removal to confirm the model’s prediction. Every design was carried out in 3 replicates, and the mean value was reported.

The experiment was carried out in 50 mL Falcon tubes by transferring 250 mg of the oven-dried FWB previously fermented by the fungal isolate (as mentioned before) for 2, 4, or 6 days (according to the experiment design) and 25 mL of dye solution. Then, all tubes were shaken at 150 rpm and 25 °C using an orbital shaker. A stock solution of 1000 mg L^−1^ Congo red in distilled water was used to prepare the required dye concentrations; 0.1 N solutions of HCl or NaOH were used to adjust the pH. The dye removal percentage as the response variable was measured at contact times of 12 and 24 h, as mentioned before [53].

### 2.6. Characterization of Wheat Bran 

Wheat bran before and after fermentation was analyzed using Fourier transform infrared spectroscopy (FTIR; Thermofisher Nicolet IS50, Waltham, MA, USA) to detect the functional groups involved in the adsorption process. IR spectroscopy is a useful technique for investigating changes in the constituent structure of lignocellulosic materials [59]. The samples were prepared as potassium bromide pellets and FTIR spectra were measured in the range of 4000–400 cm^−1^. X-ray diffraction (XRD; Bruker D8 Advance) at U = 40 kV and 1 = 30 mA was used to examine the crystalline structures, and scanning electron microscopy (SEM; Thermofisher Quanta 250FEG) was employed to study the surface morphology of wheat bran before and after fermentation. 

### 2.7. Dye Removal Assay in Liquid Cultures

An additional experiment was performed to examine the impact of different dye concentrations on dye removal efficiency by *Lasiodiplodia* sp. YZH1 in the dye-containing liquid culture; one mycelial agar disk was inoculated at a quantity of 50 mL in an Erlenmeyer flask containing 25 mL of the basal media ((NH_4_)_2_ SO_4_,1.4 g L^−1^; Na_2_HPO_4_·12H_2_O, 1.2 g L^−1^; KH_2_PO_4_, 0.9 g L^−1^; KCl, 0.5 g L^−1^; MgSO_4_·7H_2_O, 0.5 g L^−1^; yeast extract, 0.5 g L^−1^; and glucose, 1.0 g L^−1^) and Congo red in concentrations of 50, 150, and 250 mg L^−1^. Then, it was incubated at 28 °C and 150 rpm in an orbital shaker incubator. The dye removal percentage was assessed spectrophotometrically after 2, 4, 6, and 8 days of incubation.

## 3. Results and Discussion

### 3.1. Isolation and Identification of the Fungal Isolate

The morphological characteristics of the fungal isolate indicate that it is a fast-growing fungus with abundant aerial mycelium which covers the whole Petri dish area (56.75 cm^2^) in just 3 days on PDA. The initial color of the mycelium is white, then it turns smoke-gray and produces pycnidia with age. 

**Figure 1:**
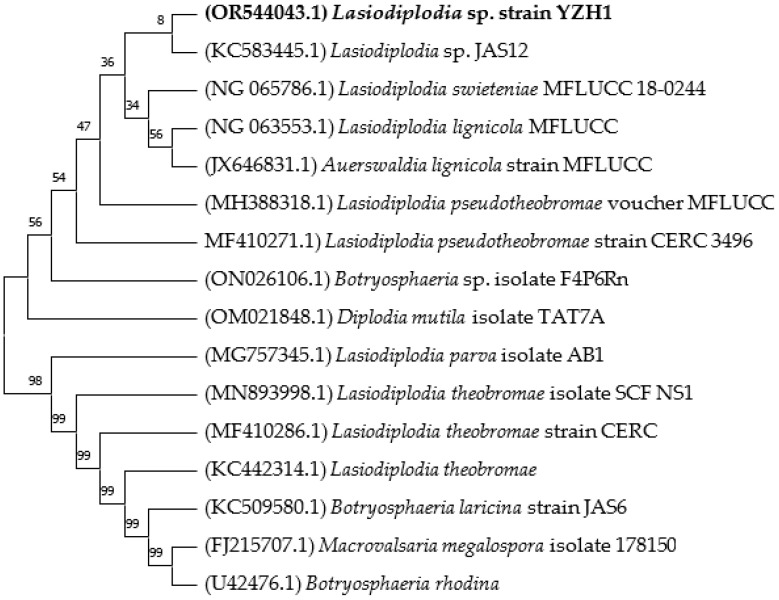
Phylogenetic tree of *Lasiodiplodia* sp. strain YZH1 with related fungal strains. The tree was constructed using the neighbor-joining method with 1000 replicates of bootstrap values. The accession numbers are given in brackets.

After comparison with other 18S rRNA sequences in the NCBI database using BLAST search, the sequencing results revealed that this strain belongs to *Lasiodiplodia* sp. YZH1 (Figure 1). The sequences were deposited in NCBI GenBank under the accession number OR544043. 

Li et al. studied the endophytic microorganisms associated with the fruiting bodies of *Tricholoma matsutake*, and revealed that they have 13 fungal strains [60]. *Calcarisporium arbuscula* is a mushroom endophytic fungus, which primarily produces the aurovertin antibiotic [61]. This is the first report isolation of an endophytic fungus *Lasiodiplodia* from the white rot fungus *Schizophyllum*. Earlier reports demonstrated the capability of the fungus. 

*Lasiodiplodia* can be used to produce ligninolytic enzymes, such as laccase and MnP. and for applications in the bioremediation of different contaminants. *Lasiodiplodia* isolated from biota containing a high concentration of xenobiotics exhibited the ability to degrade malachite green [45]. The endophytic fungus strain *Lasiodiplodia* sp. MXSF31 isolated from oleracea showed the ability to remove multiple heavy metals (Cd-, Pb-, and Zn) from contaminated water and soils [46].

### 3.2. Fungal Growth, Enzyme Assay, and Biomass Loss during SSF of Wheat Bran

**Figure 2:**
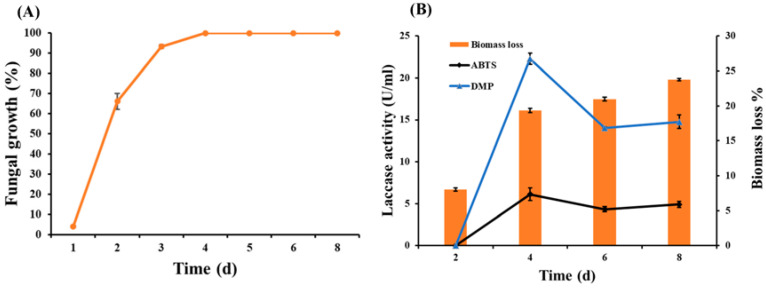
Growth curve of *Lasiodiplodia* sp. YZH1 in wheat bran (**A**) and laccase activity and biomass loss during SSF of WB with *Lasiodiplodia* sp. YZH1 (**B**) in a period of 8 days.

Wheat bran was selected in this work for solid-state fermentation with Lasiodiplodia sp. YZH1. As shown in Figure 2A, the initial mycelium production signal was observed after one day of cultivation, then the surface growth reached the maximum and stopped on the fourth day due to lack of dish area.

Due to the rapid growth of the fungi during cultivation, the biomass of the substrate was continuously consumed and considerably decreased, reaching the highest biomass loss of 23.7% after 8 days of culture (Figure 2B). The continuous loss of the substrate biomass despite the mycelium growth stopped after four days due to lack of area; this can be attributed to the three-dimensional growth of the fungus, while the fungal growth was recorded as fungal growth area (%) through the relationship between the mycelium surface area and the Petri dish surface area. This is similar to results with peanut shell fermented with white rot fungus *Pycnoporus* SYBC-L3, which achieved 25% biomass loss but after 25 days of cultivation [48].

The pH of the wheat bran continuously increased during the fermentation, reaching 5.2 on the eighth day (Supplementary Figure S1). Khaled et al. indicated that *Bacillus cereus* and *Pseudomonas parafulva* had maximum laccase production in alkaline conditions [62]. 

Laccase was produced throughout the cultivation, reaching its peak activities of 6.2 and 22.3 U/mL for the substrates ABTS and DMP, respectively, on the fourth day of cultivation (Figure 2B). The difference between these two values is due to the different type of substrate, including their different molar absorptivity coefficient and different wavelength at which each substrate absorbs.

Laccase activity expressed by *Lasiodiplodia* sp. YZH1 under SSF conditions of WB (6.2 U/mL) was higher than that expressed by *Trametes villosa* in SSF of wood chip biochar (0.7 U/mL) [49] and *Trametes* sp. in SSF of cassava residue (0.15 U/mL) [63]. On the contrary, it was lower than laccase produced by *Trametes pubescens* under semi-SSF conditions of sunflower seed shells (30.27 U/mL) [64] and *Trametes versicolor* in SSF of corncobs (911 U/mL) [65]. 

Enzyme production and biomass loss could vary considerably depending on the use of a culture substrate with a different biomass structure as well as the use of a different fungal strain with different abilities for biomass degradation.

### 3.3. Dye Removal Efficiency

**Figure 3:**
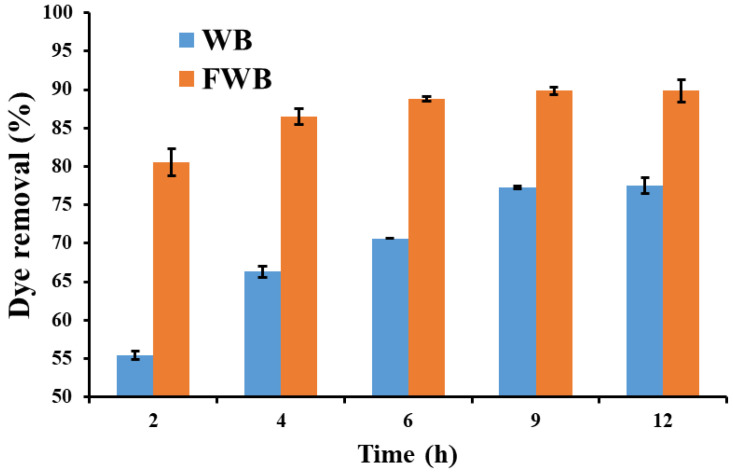
Dye removal efficiency by WB (wheat bran) and FWB (fermented wheat bran with *Lasiodiplodia* sp. YZH1).

The Congo red (100 mg L^−1^) removal assay by 6-day-fermented wheat bran (FWB) in comparison with unfermented wheat bran (WB) is shown in Figure 3.

It can be observed that Congo red removal by FWB increased sharply within the first 2 h of adsorption (80.5%), probably because there are several binding sites and enough surface area, and then after 9 h, it remained relatively constant because the available free active sites were saturated by dye molecules. Then it reached its peak (89.8%) after 12 h. On the other hand, WB removed about 55.4% of the dye in the first 2 h, reaching the maximum of 77.5% after 12 h.

These findings revealed that the dye removal efficiency of the fermented wheat bran with the fungal isolate *Lasiodiplodia* sp. YZH1 (FWB) was remarkably higher than that of unfermented wheat bran (WB), which is likely due to the fact that the fungal growth changed the physicochemical properties of the substrate (wheat bran), as well as increased the available binding active sites on the surface of the mycelium.

Similar results were obtained by Li and Jia, who recorded a maximum dye decolorization of 89.71% by *Schizophyllum* sp. under SSF of rice hull after 41 h [66]. In other reports, T. *versicolor* decolorized 86% of Astrazon Black dye adsorbed onto wheat bran under SSF conditions [67], and wheat straw and corncob shreds removed 70–75% of the color from dye solutions [68].

### 3.4. Optimization of Dye Removal

The Box–Behnken design (BBD) was applied in this study to determine the interactive effects between the three independent variables—fermentation duration of wheat bran by the fungal strain (X_1_), pH of the solution (X_2_), and dye concentration (X_3_)—and to predict the optimal conditions for dye removal. The experimental design generated by the Design-Expert^®^ software had a total of 15 runs (carried out in triplicate), as displayed in Table 2.

As can be seen, the dye removal percentage obtained from this study was in the range of 75.35–90.26% and 83.66–93.07% for the contact time of 12 and 24 h, respectively. The highest dye removal percentages of 90.26% and 93.07% were recorded in run no. 12, while run no. 6 showed the lowest value of dye removal percentages (75.35 and 83.66%) for 12 and 24 h, respectively. It can also be seen that the predicted dye removal values are quite similar to the experimental values, which indicates the model’s accuracy in predicting dye removal.

According to analysis of variance (ANOVA) analysis, the following second-order polynomial quadratic model equations for dye removal were derived:

Y_1_ = 87.83 + 3.89 X_1_ + 0.6446 X_2_ − 3.2 X_3_ − 0.1372 X_1_ X_2_ + 1.54 X_1_ X_3_ + 0.3217 X_2_ X_3_ − 1.61 X_1_^2^ − 0.5484 X_2_^2^ − 2.24 X_3_^2^ (4)

Y_2_ = 89.87 + 2.58 X_1_ + 0.7925 X_2_ + 1.56 X_3_ + 0.2198 X_1_ X_2_ + 1.74 X_1_ X_3_ + 0.9673 X_2_ X_3_ − 0.5967 X_1_^2^ − 0.2843 X_2_^2^ − 3.12 X_3_^2^ (5)

where Y_1_ and Y_2_ are the predicted dye removal percentage (%) after contact time of 12 and 24 h, respectively, X_1_ is the fermentation duration (day), X_2_ is the solution pH, and X_3_ is the dye concentration (mg L^−1^). 

The analysis of variance (ANOVA) for the quadratic prediction models (presented in Table 3 and Table 4) revealed that both models are significant for predicting the dye removal, as evidenced by the model’s F-value and low *p*-value (*p* < 0.05).

The *p*-values of both models are very low (<0.0001 and 0.0005 for 12 and 24 h, respectively), which indicates that both models are highly significant. The analysis also indicated that the lack of fit values was not significant in either model. The lack of fit of both models was 1.689 and 3.873 (for 12 and 24 h, respectively), with a *p*-value < 0.05. The non-significant lack of fit implies that the model is fit [69].

The coefficient of determination (R^2^) value was 0.9940 and 0.9854 (for Equations (1) and (2), respectively); a higher R^2^ value indicates that the model describes the data more effectively. The predicted R^2^ of 0.9142 and 0.8225 is in reasonable agreement with the adjusted R^2^ of 0.9833 and 0.9592 for models 1 and 2, respectively, proving the generated model’s accuracy in predictions.

The Adeq Precision metric measures the signal-to-noise ratio. A ratio greater than 4 is desirable. The present ratios are 33.4159 and 18.1209, indicating that the signal is strong enough to be used to navigate the design space.

*p*-values are also used to find out the significance of each variable, which in turn helps in understanding the pattern of interaction between the tested factors. It is obvious from the data that the linear effect of all variables (X_1_, X_2_, X_3_) was significant (*p* < 0.05) in the dye removal. The interaction effect between X_1_X_3_ on the dye removal was significant, while the interactions between X_1_X_2_ were not significant.

### 3.5. Validation of the Design Model 

The selected quadratic model was laboratory-validated by conducting an additional experiment at 6 days of wheat bran fermentation duration, 8.265 of pH dye solution, and 164.88 mg/L of dye concentration. The obtained results (Table 5) were 89.485 and 93.402% after a contact time of 12 and 24 h, respectively. These values came within the confidence interval as specified by the model, which confirms the accuracy of the model.

**Figure 4:**
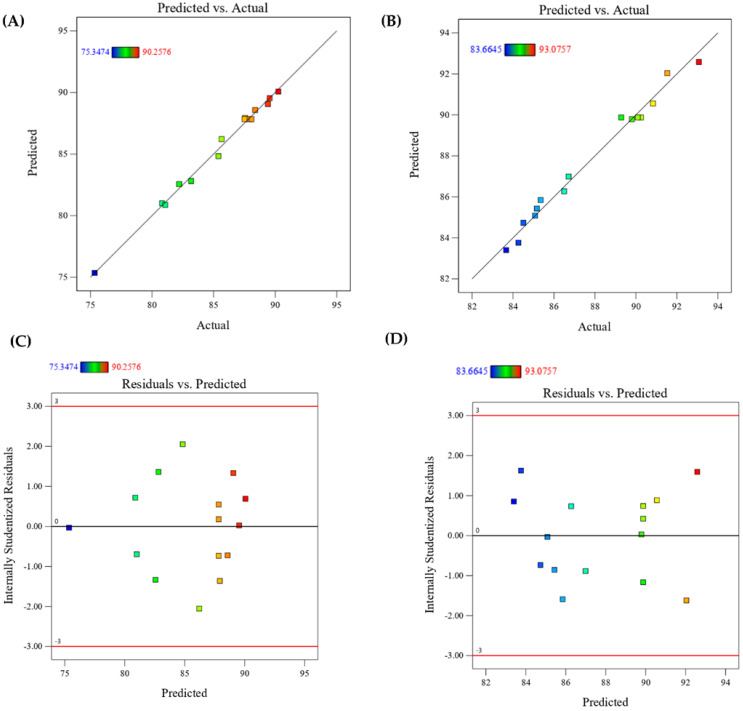
The parity plots illustrate the relationship between experimental and predicted values after contact time of 12 h (**A**) and 24 h (**B**) and residual versus predicted response values after 12 h (**C**) and 24 h (**D**).

The actual values were plotted against predicted values in Figure 4A,B. It can be clearly seen that the actual and predicted values are linearly correlated, where the correlation coefficients are 0.997 and 0.992 for the dye removal percentage after the contact time of 12 and 24 h, respectively. The points are either on or very close to the 45-degree straight line, indicating that the residuals are normally distributed and the response surface model is accurate. This suggests that the experimental points are well-aligned with the predicted values and the quadratic model fits the data well.

Figure 4C,D show the residuals versus fitted response values (predicted). The residuals are randomly distributed, as the number of points above and below the horizontal line is approximately equal. Additionally, the residual values are within the range of ±3.00, which is considered a threshold for identifying outliers [70].

Three-dimensional (3D) response surface plots were created to illustrate the interactive effects of the three variables on the dye removal efficiency and the optimal value of each variable required to maximize dye removal %.

**Figure 5:**
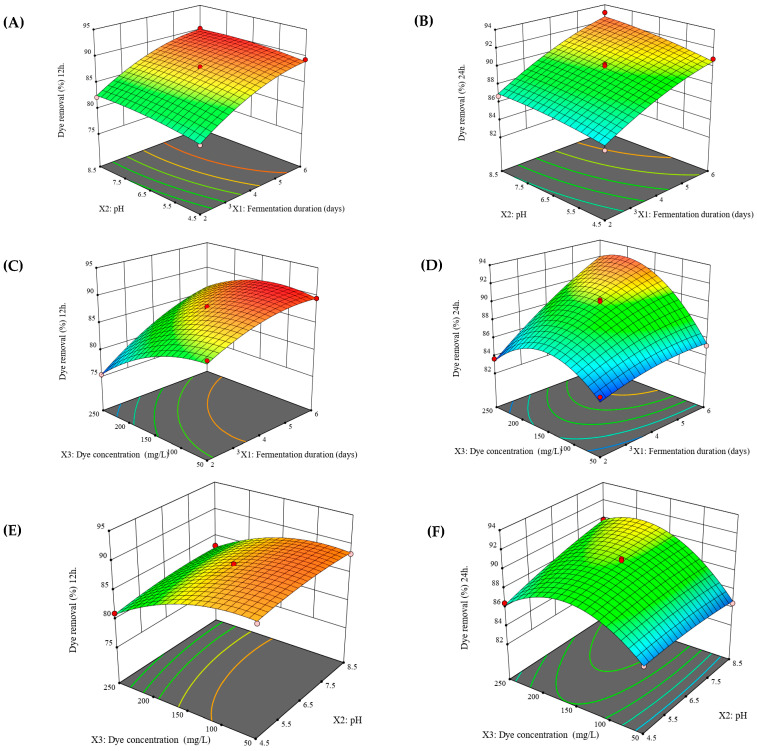
Three-dimensional response surface plots showing the interactive effects between each of the two variables on dye removal efficiency by FWB after contact time of 12 h (**A**,**C**,**E**) and after contact time of 24 h (**B**,**D**,**F**).

Figure 5 illustrates 3D surface plots of the interaction effect of each two variables on the dye removal by FWB after contact time of 12 and 24 h, while the third variable was set at the center point (level 0). 

The simultaneous effect of wheat bran fermentation duration (X_1_) and pH of the solution (X_2_) on dye removal efficiency after 12 and 24 h, with the initial dye concentration (X_3_) fixed at the central point of 150 mg/L, is shown in Figure 5A,B. It can be seen that an increase in both fermentation duration and pH of the solution led to a significant increase in dye removal. Figure 5C,D show the interaction effect of fermentation duration and initial dye concentration on the dye removal. An increase in fermentation duration led to a significant increase in dye removal, and the interaction between these two factors had a highly significant effect (*p*-value < 0.05) on the dye removal efficiency.

Accordingly, the fermentation duration of wheat bran by the fungal strain plays an important role in dye removal percentage. As the fermentation period increases, the fungus growth in wheat bran increases. There are no data available in the literature about the effect of fermentation duration of the adsorbent on dye removal efficiency; however, this positive effect can mostly be attributed to the increase in mycelium content and the increase in the numbers of binding active sites as well as available surface area of the adsorbent as a result of increased fungal growth with increased fermentation duration, which was confirmed by FTIR, XRD, and SEM analysis. 

The effect of pH and initial dye concentration on dye removal is displayed in Figure 5E,F. It’s obvious that, solution pH had a slight effect on dye removal, while the initial dye concentration had a significant effect on dye removal efficiency. An increase in dye concentration led to a significant decrease in dye removal, especially after a contact time of 12 h. 

Tejada-Tovar et al. reported, at higher initial dye concentrations, the available active sites on the adsorbent are limited, which in turn lead to a reduction in the dye removal percentage [71]. Similar results were obtained by Arellano G. Rodríguez et al. who noted a negative correlation between removal efficiency and dye concentration occurred during Congo red removal by cocoa bean shells [72]. 

However, after the contact time of 24 h, the removal efficiency at first increased as the dye concentration increased, and then declined at higher dye concentrations (>150 mg L^−1^). This pattern of dye removal is also similarly reported by [73,74].

The maximum predicted dye removal of 89.877 and 93.076% after a contact time of 12 and 24 h, respectively, were achieved at 6 days fermentation duration, 8.266 pH and an initial dye concentration of 164.871 mg/L.

### 3.6. Characterization of Wheat Bran 

FTIR spectroscopy was employed to analyze wheat bran before and after fermentation, as well as after dye adsorption to investigate the changes in the surface functional groups on the biosorbent.

Given that wheat bran and fungal biomass is mostly composed of polysaccharides (cellulose or glucans) and proteins, the presence of numerous functional groups on the biomass surface, including carboxyl, amine, and hydroxyl groups, could be useful for adsorption [75]. 

**Figure 6:**
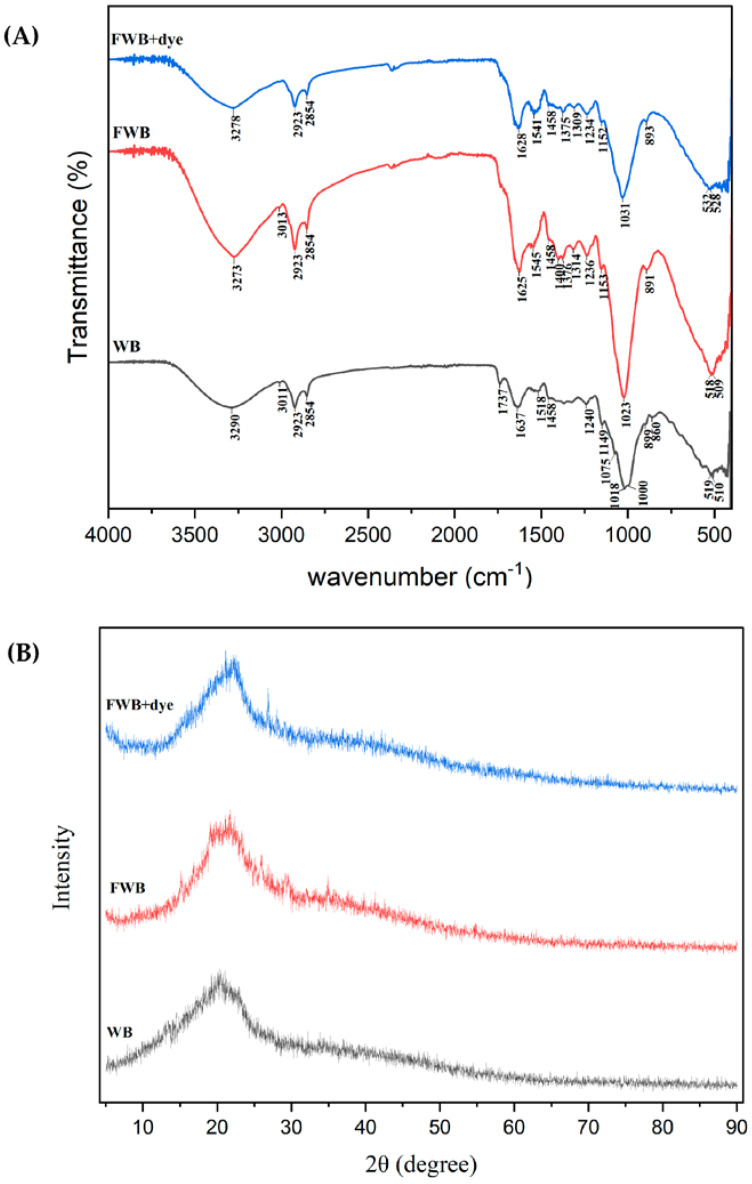
Characterization of WB and FWB: (**A**) FTIR spectra; (**B**) XRD spectra.

As seen in Figure 6A, the FTIR spectra displayed a variety of absorption peaks, indicating the complex functional sites on the surface. The absorption peaks at wave numbers of 3290, 3273, and 3278 cm^−1^ indicate that the stretching vibration of the OH functional group originated from the cellulose-derived materials [76]. The band around 1000 cm^−1^ was related to C–O–C stretching vibrations in cellulose and hemicellulose [77].

FWB showed lower transmittance at 2923 and 2854 cm^−1^ (associated with the C-H vibration) compared with the WB. Unfermented wheat bran (WB) had the functional group with a signal at 1737 cm^−1^, which was attributed to C=O stretching of carboxyl groups and ketones [78]. The transmittance at the wavelengths of 1149, 1075, 899, and 860 cm^−1^ (b-glycosidic bond vibrations, mostly in hemicelluloses) reduced after fungal fermentation, indicating that cellulose and hemicellulose were degraded [79]. 

The reduced transmittance and changes in absorption bands at wavelengths of 1637, 1518, and 1458 cm^−1^ (stretching of the C=C and C=O lignin aromatic ring) demonstrated the change in aromatic hydrocarbon compounds and lignin degradation [80,81]. IR bands near 1240 or 1737 cm^−1^ (representing acetyl groups) were used as diagnostic spectral peaks for cellulose [82]. The changes in the wave numbers lower than 1000 cm^−1^ were due to the presence of heterocyclic aromatics in lignin. This demonstrates that the functional sites on wheat bran were somewhat altered by fungus treatment [48].

After fermentation, the peaks at 1737, 1075, and 860 cm^−1^ (associated with carbon bonds) disappeared, proving that the depolymerization process led to chemical changes in the wheat bran structure. Furthermore, some peaks displayed higher intensity after fermentation, such as those at 3273, 2923, 1625, 1236, and 1023 cm^−1^ (related to O-containing groups). The appearance of new peaks at 1400 cm^−1^ (associated with carboxylate group), 1376 (C–H bending of amorphous cellulose), and 1314 (C–H bending vibration in crystallized cellulose) proves that fungal fermentation alters the wheat bran structure of cellulose. 

As for the spectrum after dye adsorption, by comparing with the wavenumbers of the peaks and its intensity before and after dye adsorption, it can be clearly seen that some peaks shifted, while others weakened or enhanced [83]. The change in peak wave number after dye adsorption demonstrates its involvement in the reaction (biosorption), while the weakened intensity of a functional group implies a reduction in its content [80].

The chart of analysis shows that the absorption peak of the stretching vibration of the OH at 3273 weakened and shifted to 3278 after adsorption, indicating its participation in the dye removal.

Similarly, the peaks at 1625 and 1545 (amide group vibrations) weakened and shifted to 1628 and 1541, respectively, the peak at 1376 (C–H bending of cellulose) shifted to 1375, and 1314 (C–H bending vibration) shifted to 1309 cm^−1^ and 1236 to 1234 (C–O–C) after dye adsorption. Also, it can be clearly seen that the strong absorption peak at 1023 weakened and shifted to 1031 (related to S=O stretching vibrations), demonstrating the existence of Congo red on the biosorbent after dye adsorption, since this chemical bond corresponds to Congo red structure.

The disappearance of the peak at 1400 (related to carboxylate group) confirmed the involvement of the functional group in the dye adsorption.

The XRD patterns of wheat bran before and after fermentation and after dye adsorption are shown in Figure 6B. The XRD pattern showed a major broad peak between 10° and 30°, representing the cellulose diffraction peak. The two minor peaks in XRD spectrum fungal fermentation at 26° and 29° are possibly related to the low degree structure of polysaccharides [80,83].

The absence of sharp peaks demonstrates the non-crystalline nature of the wheat bran or fermented wheat bran and supports their amorphous structures [49,84]. These disordered structures contain several functional groups on the absorbent surface, such amide, carboxyl, and phosphate, which enhance the dye adsorption process [49,85]. A similar structure pattern was also found in *Pleurotus ostreatus* spent substrate [83], *Beauveria bassiana* [86], *Agaricus bisporus* residue [84], nutshell biochar [87], microalgae biochar [88], and wood chip biochar [49].

SEM micrographs were used to compare the surface morphology of wheat bran before and after fermentation. 

**Figure 7:**
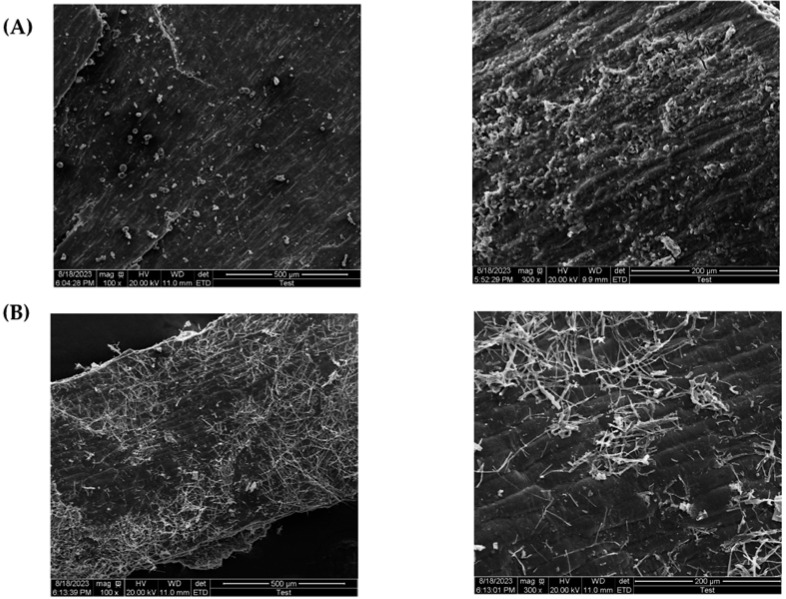
Scanning electron microscopy (SEM) images of WB (**A**); FWB (**B**) at magnifications of ×100 and ×300.

As shown in Figure 7, unfermented wheat bran has starch granules and adherent endosperm protein on its surface [89], while after fermentation, they were consumed by the fungi. Additionally, after fungal fermentation, the wheat barn surface was colonized by the fungal mycelium and made the fibrous cellulose more exposed, with an irregular surface, resulting in increasing external surface area [81]. These features of the biosorbent led to a high adsorption capacity [49]; hence, it can be said that wheat bran fermentation by *Lasiodiplodia* sp. YZH1 enhanced its adsorption efficiency.

### 3.7. Dye Removal Assay in Liquid Cultures

An additional experiment was conducted to investigate the dye removal efficiency by the fungal strain at three dye concentrations (50, 150, 250 mg L^−1^).

**Figure 8:**
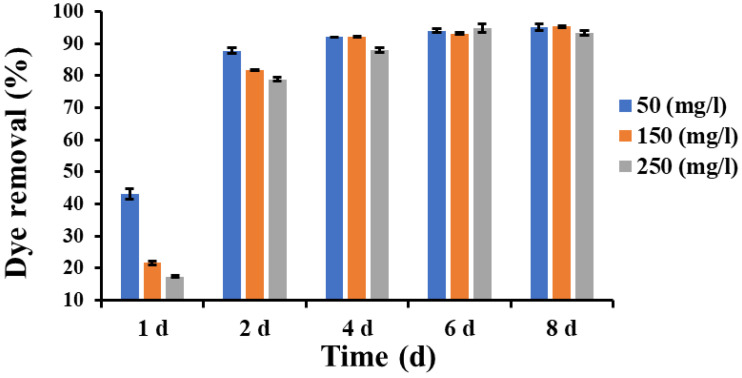
Dye removal efficiency by *Lasiodiplodia* sp. YZH1 in 50, 150, 250 mg L^−1^ dye-containing liquid cultures.

Figure 8 shows that the removal percentage after 1 day of inoculation was 43.2, 21.6, and 17.4%, then it increased sharply after 2 days to become 87.8, 81.7, and 78.8% for the dye concentrations of 50, 150, and 250 mg L^−1^, respectively. The decrease in dye removal efficiency at higher dye concentrations was probably caused by the saturation of the fixed number of biosorbent active sites.

From 4 to 8 days, the dye removal percentage increased slightly and reached the highest values of 95% and 95.3% for the concentrations of 50 and 150 mg L^−1^, respectively, and it reached 94.8% for the concentration of 250 mg L^−1^ after 6 days of inoculation.

These results indicated the ability of the fungal strain *Lasiodiplodia* sp. YZH1 to remove Congo red dye from aqueous solutions, which occurs primarily via an adsorption process on the fungal biomass present in the culture. Similar results were obtained by Asses et al. 2018, who recorded 97% decolorization of Congo red (200 mg L^−1^) by *Aspergillus niger* after 6 days at 120 to 150 rpm shaking speed [22].

## 4. Conclusions

This study reported the effectiveness of SSF of wheat bran with endophytic fungi *Lasiodiplodia* isolated from white rot fungus *Schizophyllum* for the first time to decolorize Congo red azo dye from aqueous solutions, as well as the ability of this fungal strain to remove Congo red dye in liquid culture. The fermented wheat (FWB) bran removed 89.8% of the dye (100 mg L^−1^), whereas WB removed only 77.5% after 12 h of contact with the dye solution. According to the Box–Behnken design results, FWB removed 93.08% of the dye under the condition of 6 days’ fermentation duration, and resulted in pH 8.5 and 150 mg L^−1^ dye concentration after 24 h. Additionally, the fungal strain removed 95.3% of the dye (150 mg L^−1^) in liquid culture after 8 days of inoculation. Ultimately, this study introduced a cheap, effective, and environmentally friendly procedure for biodecolorization of dye from aqueous solutions. In future studies, this fungal strain needs to be applied for industrial wastewater treatment on a larger scale and in bioreactor conditions, and SSF also should be performed with other agro-industrial wastes in order to assess its ability for bioremediation of other xenobiotics.

## Figures and Tables

**Table 1:** Ranges and levels of the Box–Behnken design.

Variable		Range and Level		
Name	Unit	−1	0	1
Wheat bran fermentation duration (X_1_)	day	2	4	6
pH of the dye solution (X_2_)	−	4.5	6.5	8.5
Dye concentration (X_3_)	mg L^−1^	50	150	250

**Table 2:** Box–Behnken experimental design matrix for optimization of dye removal by solid-state fermentation of wheat bran with *Lasiodiplodia* sp. YZH1 (FWB).

Run	Variables			Response 1 Dye Removal Percentage after 12 h (%)		Response 2 Dye Removal Percentage after 24 h (%)	
	Fermentation Duration (X_1_)	Solution pH (X_2_)	Dye Concentration (X_3_)	Experimental	Predicted	Experimental	Predicted
1	6	6.5	50	89.55	89.54	85.17	85.43
2	4	6.5	150	87.51	87.83	89.28	89.87
3	4	4.5	50	87.56	87.93	85.07	85.08
4	4	6.5	150	87.91	87.83	90.25	89.87
5	2	8.5	150	82.21	82.57	86.71	86.99
6	2	6.5	250	75.35	75.35	83.66	83.40
7	4	8.5	50	88.38	88.57	84.51	84.73
8	6	4.5	150	89.41	89.06	90.83	90.56
9	2	4.5	150	80.81	81.00	85.35	85.84
10	2	6.5	50	85.39	84.84	84.27	83.76
11	4	6.5	150	88.08	87.83	90.09	89.87
12	6	8.5	150	90.26	90.07	93.08	92.58
13	4	8.5	250	83.18	82.81	89.80	89.79
14	4	4.5	250	81.07	80.88	86.50	86.27
15	6	6.5	250	85.66	86.21	91.53	92.04

**Table 3:** Analysis of variance (ANOVA) for the Box–Behnken design experimental outcomes of dye removal after 12 h of adsorption by FWB.

Source	Sums of Squares	df	Mean Square	F-Value	*p*-Value	Remarks
Model	242.83	9	26.98	92.75	<0.0001	Significant
X_1_	121.05	1	121.05	416.13	<0.0001	Significant
X_2_	3.32	1	3.32	11.43	0.0197	Significant
X_3_	82.07	1	82.07	282.12	<0.0001	Significant
X_1_X_2_	0.0753	1	0.0753	0.2588	0.6326	Not significant
X_1_X_3_	9.46	1	9.46	32.52	0.0023	Significant
X_2_X_3_	0.4139	1	0.4139	1.42	0.2865	Not significant
X_1_^2^	9.59	1	9.59	32.97	0.0022	Significant
X_2_^2^	1.11	1	1.11	3.82	0.1082	Not significant
X_3_^2^	18.46	1	18.46	63.47	0.0005	Significant
Residual	1.45	5	0.2909			
Lack of Fit	1.29	3	0.4286	5.08	0.1689	Not significant
Pure Error	0.1688	2	0.0844			
Cor Total	244.29	14				
Std. Dev.	0.5394	R^2^ =		0.9940		
Mean	85.49	Adj. R^2^ =		0.9833		
C.V. %	0.6309	Pred. R^2^ =		0.9142		
PRESS	20.95	Adeq. Precision		33.4159		

Std. Dev.: standard deviation; C.V.: coefficient of variation; PRESS: prediction error sum of squares; Adeq. Precision: adequate precision.

**Table 4:** Analysis of variance (ANOVA) for the Box–Behnken design experimental outcomes of dye removal after 24 h of adsorption by FWB.

Source	Sums of Squares	df	Mean Square	F-Value	*p*-Value	Remarks
Model	130.11	9	14.46	37.53	0.0005	Significant
X_1_	53.10	1	53.10	137.83	<0.0001	Significant
X_2_	5.02	1	5.02	13.04	0.0154	Significant
X_3_	19.48	1	19.48	50.56	0.0009	Significant
X_1_X_2_	0.1932	1	0.1932	0.5016	0.5104	Not significant
X_1_X_3_	12.14	1	12.14	31.53	0.0025	Significant
X_2_X_3_	3.74	1	3.74	9.72	0.0263	Significant
X_1_^2^	1.31	1	1.31	3.41	0.1240	Not significant
X_2_^2^	0.2984	1	0.2984	0.7746	0.4191	Not significant
X_3_^2^	35.96	1	35.96	93.33	0.0002	Significant
Residual	1.93	5	0.3852			
Lack of Fit	1.39	3	0.4632	1.73	0.3873	Not significant
Pure Error	0.5367	2	0.2683			
Cor Total	132.04	14				
Std. Dev.	0.5394	R^2^ =		0.9940		
Mean	85.49	Adj. R^2^ =		0.9833		
C.V. %	0.6309	Pred. R^2^ =		0.9142		
PRESS	20.95	Adeq. Precision		33.4159		

Std. Dev.: standard deviation; C.V.: coefficient of variation; PRESS: prediction error sum of squares; Adeq. Precision: adequate precision.

**Table 5:** Validation of the design model.

Analysis	Predicted Mean	Predicted Median	Std Dev	SE Pred	95% PI Low	Data Mean	95% PI High
12 h	89.877	89.877	0.539	0.689	88.106	89.485	91.648
24 h	92.882	92.882	0.594	0.713	91.138	93.402	95.114

## Data Availability

Data are contained within the article.

## Supplementary Materials

The following supporting information can be downloaded at: https://www.mdpi.com/article/10.3390/jof9111069/s1, Figure S1: pH of the fermented wheat bran with *Lasiodiplodia* sp. YZH1 under SSF condition at a period of 8 days.
