# Supplementary material for: Decolorization of Textile Azo Dye via Solid-State Fermented Wheat Bran by Lasiodiplodia sp. YZH1
Source: J Fungi (Basel). 2023 Nov 1;9(11):1069. doi: 10.3390/jof9111069 (PMC10672102; doi:10.3390/jof9111069)
Supplement: Supplementary file 1 [file jof-09-01069-s001.zip › jof-2667027-supplementary.pdf]

Decolorization of Textile Azo Dye via Solid-State Fermented Wheat Bran by *Lasiodiplodia* sp. YZH1

Ali Borham [1,2,3], Mohammad K. Okla[4], Mohamed A. El-Tayeb[4], Ahmed Gharib[5], Hanan Hafiz[6], Lei Liu[7], Chen Zhao[7], Ruqing Xie[7], Nannan He[7] , Siwen Zhang[7], Juanjuan Wang[1,7] and Xiaoqing Qian[1,7,*]

[1] Key Laboratory of Cultivated Land Quality Monitoring and Evaluation, Ministry of Agriculture and Rural Affairs, Yangzhou University, Yangzhou 225127, China; ali.borham@agr.kfs.edu.eg (A.B.); wangjuanjuan@yzu.edu.cn (J.W.); qianxq@yzu.edu.cn (X.Q.)

[2] Agriculture Products Safety and Environment, College of Agriculture, Yangzhou University,
Yangzhou 225127, China

[3] Agricultural Botany Department, Faculty of Agriculture, Kafrelsheikh University, Kafr El-Sheikh 33516, Egypt

[4] Botany and Microbiology Department, College of Science, King Saud University, P.O. Box 2455,
Riyadh 11451, Saudi Arabia; malokla@ksu.edu.sa (M.K.O.); mali5@ksu.edu.sa (M.A.E.-T.)

[5] National Institute of Laser Enhanced Sciences (NILES), Cairo University, Giza 12613, Egypt; ahmgharib@niles.edu.eg (A.G.)

[6] Biotechnology Department, Faculty of Science, Damietta University, New Damietta 34517, Egypt; hananhafizz5@gmail.com (H.H.)

[7] College of Environmental Science and Engineering, Yangzhou University, Yangzhou 225127, China; ll18252788565@163.com (L.L.); zhaochen1117yz@163.com (C.Z.); 15252753630@163.com (R.X.); hnn991012@163.com (N.H.); zhangsiwen980202@163.com (S.Z.)

* Correspondence: qianxq@yzu.edu.cn

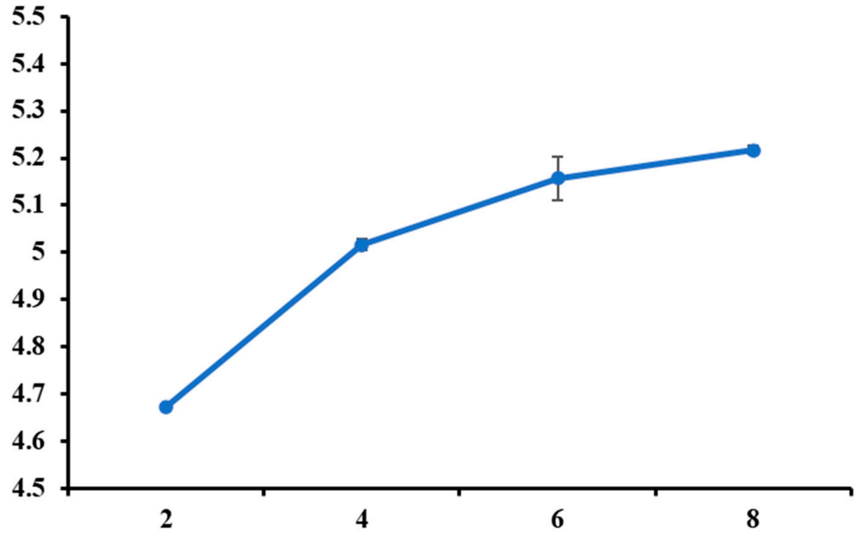

Figure S1. pH of the fermented wheat bran with *Lasiodiplodia* sp. YZH1 under SSF condition at a period of 8 days.
